# Imaging of tumor colonization by *Escherichia coli* using ^18^F-FDS PET

**DOI:** 10.7150/thno.42121

**Published:** 2020-04-01

**Authors:** Sae-Ryung Kang, Eui Jeong Jo, Vu Hong Nguyen, Ying Zhang, Hee Seung Yoon, Ayoung Pyo, Dong-Yeon Kim, Yeongjin Hong, Hee-Seung Bom, Jung-Joon Min

**Affiliations:** 1 Department of Nuclear Medicine, Chonnam National University Medical School and Hwasun Hospital, Hwasun, Jeonnam, Korea; 2 Institute for Molecular Imaging and Theranostics, Chonnam National University Medical School, Hwasun, Jeonnam, Korea; 3 Department of Experimental Therapeutics, Beckman Research Institute of City of Hope, Duarte, CA 91010, USA; 4 Department of Microbiology, Chonnam National University Medical School, Hwasun, Jeonnam, Korea

**Keywords:** tumor-targeting bacteria, *Escherichia coli*, bacterial cancer therapy, ^18^F-fluorodeoxysorbitol (^18^F-FDS), positron emission tomography (PET)

## Abstract

Tumor-targeting bacteria have been actively investigated as a new therapeutic tool for solid tumors. However, *in vivo* imaging of tumor-targeting bacteria has not been fully established. ^18^F-fluorodeoxysorbitol (FDS) positron emission tomography (PET) is known to be capable of imaging Gram-negative Enterobacteriaceae infection. In the present study, we aimed to validate the use of ^18^F-FDS PET for visualization of the colonization and proliferation of tumor-targeting *Escherichia coli* (*E. coli*) MG1655 in mouse tumor models.

**Methods:** *E. coli* (5 × 10^7^ colony forming unit) were injected intravenously into BALB/c mice bearing mouse colon cancer (CT26). Before and 1, 3, and 5 days after the bacterial injection, PET imaging was performed following i.v. injection of approximately 7.4 MBq of ^18^F-FDS. Regions of interest were drawn in the engrafted tumor and normal organs including the heart, liver, lung, brain, muscle, and intestine. Semiquantitative analysis was performed using maximum standardized uptake value (SUV_max_).

**Results:** ^18^F-FDS uptake was significantly higher in tumors colonized by live *E. coli* MG1655 than in uncolonized tumors (*p* < 0.001). The PET signals in the colonized tumors at 3 days after bacterial injection were 3.1-fold higher than those in the uncolonized tumors. Tumoral ^18^F-FDS uptake correlated very strongly with the number of *E. coli* in tumors (r = 0.823, *p* < 0.0001). Cross sectional analysis of autoradiography, bioluminescence, and pathology revealed that the ^18^F-FDS uptake sites in tumors matched the locations of *E. coli* MG1655.

**Conclusion:** In conclusion, ^18^F-FDS PET is expected to be useful for the semiquantitative visualization of tumor-targeting bacteria when bacterial cancer therapy is performed using Gram-negative Enterobacteriaceae such as *E. coli*.

## Introduction

Current cancer therapies often encounter challenges, including the nonspecific systemic distribution of antitumor agents, inadequate drug concentrations in the tumor, refractoriness in hypoxic tumor, intolerable cytotoxicity, and the development of multiple drug resistance. Bacterial cancer therapy (BCT) offers a number of unique features that can help to achieve anticancer therapeutics with a desired level of consistency; it can specifically target tumor tissue, it can destroy hypoxic tumor as well as normoxic tumor while minimizing damage to normal cells, and the bacteria can self-proliferate to reach an adequate density 1, 2. The number of published BCT studies has increased exponentially, driven almost entirely by the increasing use of Gram-negative Enterobacteriaceae such as *Salmonella* and *Escherichia coli* (*E. coli*) as the drug delivery systems 1. In addition, with the recent developments in synthetic biology, preclinical studies using engineered *Salmonella* and *E. coli* have shown improved therapeutic effects in tumor models 3-5. Recently, clinical trials have commenced with several tumor-targeting bacterial strains including Gram-negative (*Salmonella* 6, 7) and Gram-positive (*Clostridium* 8, 9 and *Bifidobacterium* 10) bacteria in patients with advanced and refractory solid tumors. Several studies using *Clostridium* have yielded encouraging results with robust tumor colonization and tumor lysis in different cancer types 2. *Salmonella* strains also show tumor colonization and therapeutic benefit in refractory solid tumor patients 7.

Non-invasive imaging of the therapeutic process of BCT is important, not only for preclinical studies, but also for future applications in human patients. Confirmation that bacteria have successfully localized and proliferated in the tumor is important for predicting and evaluating the therapeutic effect of the bacteria. The monitoring of bacterial colonization in other organs (the off-target effect) is also important for the prediction of possible adverse events associated with infection or direct cytotoxicity to normal tissues. In preclinical studies, optical imaging techniques based on bioluminescence and fluorescence have been extensively employed to image the therapeutic process of BCT 11-15; however, because of technical limitations, it is hard to perform such optical imaging in human bodies 16. Therefore, in human studies, assessment of bacterial colonization has been done via invasive sampling of tumor tissues, collection of blood, urine, and stool samples, and observation of clinical signs of inflammation and infection.

Positron emission tomography (PET) and magnetic resonance imaging (MRI) might be strong candidates for overcoming the limitations of optical imaging, as they both show high sensitivity with an unlimited depth of penetration. PET reporter genes such as herpes simplex virus 1 thymidine kinase (HSV1-TK) 17, 18 and bacterial endogenous TK 19 have been addressed to radiolabeled nucleoside analogs. Attenuated *Salmonella typhimurium (S. typhimurium*) expressing HSV1-TK was successfully imaged with ^124^I-2'-fluoro-1-β-D-arabino-furanosyl-5-iodo-uracil (^124^I-FIAU). ^18^F-2'-Fluoro-2'deoxy-1-β-D-arabinofuranosyl-5-ethyl-uracil (^18^F-FEAU) PET was used to show that probiotic *E. coli* Nissle 1917 with endogenous TK localized in tumors. MRI has also been used with a few limited species of bacteria such as the magnetite-producing bacteria (*Magnetospirillum magneticum* AMB-1) 20 or *Clostridium novyi*-NT spores labeled with iron-oxide nanoclusters 21. However, such imaging methods require a top-down bacterial engineering approach, complicated synthesis of radiotracers, or can only be addressed in a limited range of bacterial strains. These limitations have hampered the widespread use of these imaging methods in preclinical or clinical studies.

Recently, ^18^F-fluorodeoxysorbitol (FDS) has been used to image infections of Enterobacteriaceae, a large family of Gram-negative bacteria known to use sorbitol as a metabolic substrate 22-24. In previous studies, ^18^F-FDS was shown to selectively accumulate in Enterobacteriaceae such as *E. coli*, but not in Gram-positive bacteria or healthy mammalian or cancer cells. For more than a decade, we have explored Gram-negative Enterobacteriaceae such as *E. coli* 11, 12, 25 or *Salmonella* spp. 13, 25-27 for use in BCT. Therefore, in the present study, we evaluated the use of ^18^F-FDS PET for the visualization of tumor colonization and proliferation of tumor-targeting *E. coli* in mouse tumor models.

## Materials and Methods

### Bacterial strains

Wild-type *E. coli* K-12 strain (MG1655, ATCC 700926) 11, 12 and attenuated *S. typhimurium* (14028s) defective in ppGpp synthesis (ΔppGpp *S. typhimurium*) 13 were used in the present study. *E. coli* ATCC 25922 and *Staphylococcus aureus* (*S. aureus*) strains were purchased from American Type Culture Collection (ATCC) and PerkinElmer, respectively. Bioluminescent *E. coli* was engineered as previously reported 11, 12.

### Radiosynthesis of ^18^F-FDS

**Figure 1:**
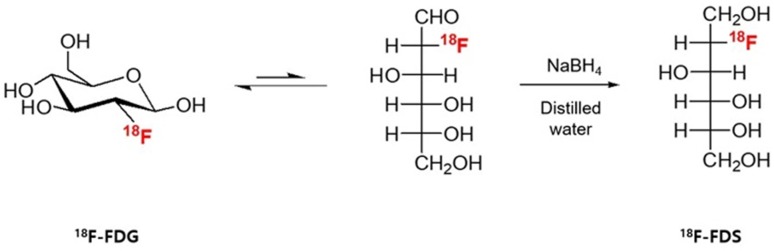
** Synthesis of ^18^F-FDS.** ^18^F-fluorodeoxysorbitol (FDS) was synthesized by reducing ^18^F-fluorodeoxyglucose (FDG) using NaBH_4_.

The ^18^F-FDS was prepared by modifying a previously reported method 22. Briefly, 2-[^18^F]-fluorodeoxyglucose (^18^F-FDG) was synthesized and provided by the Department of Nuclear Medicine at Chonnam National University Hwasun Hospital. ^18^F-FDG (555 MBq) was reduced with sodium borohydride (NaBH_4_, 2 mg, 0.053 mmol) at 45 °C for 15 min before quenching with acetic acid and pH correction to 7.4 with sodium bicarbonate (Figure 1). Finally, the solution was filtered directly into a sterile product vial through a Sep-Pak alumina N cartridge with a sterile Millipore filter (0.22 μm, 4 mm). Radiochemical purity of the ^18^F-FDS was determined by high-performance liquid chromatography.

### *In vitro* uptake test of ^18^F-FDS

The two cancer-targeting bacterial strains *E. coli* MG1655 and ΔppGpp *S. typhimurium* 26, wild-type *S. aureus*, and heat-killed (90 ℃ for 30 min) *E. coli* MG1655 and ΔppGpp *S. typhimurium* were diluted to 7 × 10^7^ colony forming units (CFU)/ml with lysogeny broth (LB) containing 0.37 MBq/ml of ^18^F-FDS. The bacteria were grown at 37 ℃ for 1 or 2 h in a shaking incubator, harvested by centrifugation (21124 ×g, 5 ℃, 7 min), and then washed three times with phosphate-buffered saline (PBS). The radioactivity in the pelleted bacteria and supernatant were measured using an automated gamma counter (Wallac Wizard 1480, PerkinElmer).

### Animal models and bacterial infection

Six-week old female BALB/c mice were purchased from the Orient Company. To prepare the tumor models, 5 × 10^6^ CT26 murine colon cancer cells were injected subcutaneously into the forelimbs of the mice. Two weeks after tumor cell inoculation, 5 × 10^7^ CFU of *E. coli* MG1655 in 100 μl PBS was injected via the tail vein. The mice were euthanized and sacrificed at 1, 3, and 5 days postinoculation (dpi). Tumor volume (mm^3^) was calculated using the formula (L × W × H)/2, where L is the length, W is the width, and H is the height of the tumor in millimeters. Tissues were removed, placed individually into sterile tubes containing PBS at 4 °C, and weighed. Samples were transferred to sterile homogenization tubes, homogenized, and then returned to their original tubes for serial dilution with PBS. LB agar plates were inoculated with diluted homogenate and incubated overnight at 37 °C. Colonies were counted and the bacterial load was expressed as CFU g-1 tissue.

All the animal experiments were conducted in accordance with the guidelines of the Animal Care and Use Committee of Chonnam National University.

### ^18^F-FDS PET imaging and image analysis

^18^F-FDS microPET imaging studies were performed before and after intravenous injection of *E. coli* MG1655. PET images of CT26-bearing BALB/c mice were obtained on a microPET scanner (Inveon, Siemens Medical Solutions, Knoxville, TN, USA) 2 h after tail vein injection of ^18^F-FDS (7.4 MBq). Static microPET images were acquired for 10 min.

For the ^18^F-FDS PET image analysis, spherical regions of interest (ROIs) were drawn in the tumors and normal organs including heart, liver, lung, intestine, brain, and thigh muscle. The maximum standardized uptake value (SUV_max_) was used for the semiquantitative analysis of ^18^F-FDS uptake, with the SUV ratio being defined as (SUV_max_ in post-bacterial injection PET)/(SUV_max_ in pre-bacterial injection PET).

### Autoradiography and optical imaging

To compare optical imaging signals with ^18^F-FDS uptake, bioluminescent bacteria were generated by transforming *E. coli* MG1655 with an expression plasmid (pLux) containing the luxCDABE operon from *Photobacterium leiognathi*, as previously described 11. Two weeks after tumor cell inoculation, four mice were intravenously injected with 7.4 MBq (200 μCi) of ^18^F-FDS before and after injection of *E. coli* MG1655 expressing Lux (1, 2, and 3 dpi, 5 × 10^7^ CFU). *In vivo* bioluminescence imaging was performed using a cooled charge-coupled device camera system (NightOWL LB 983, Berthold Technologies, Bad-Wildbad, Germany). Tumors were then removed from sacrificed mice and frozen immediately in embedding medium (Leica, USA). Serial sectioning was performed at 2 mm thickness using a slicer (Alto, 1 mm, CellPoint Scientific, USA). After acquiring bioluminescence imaging, the tumor slices were immediately transferred to a phosphor imaging plate (BAS MS 2025, FUJIFILM, Tokyo, Japan) and exposed for 24 h at -20 ℃. The resulting autoradiograms were scanned using a Typhoon FLA 9500 scanner (GE Healthcare, Uppsala, Sweden). Phosphor imaging data were analyzed using ImageQuant TL V8.1 software (GE Healthcare, Chicago, IL, USA).

### Statistical analysis

Statistical analysis was performed using GraphPad Prism 6 (GraphPad Software Inc.). The means of different groups were compared using two-tailed Student's t-test, Mann-Whitney U tests, or Kruskal-Wallis tests (for multiple comparisons). Correlations between bacterial counts and imaging signals or tumor size were performed using Spearman correlation. Data are presented as means and SEM. Differences showing a *p* value < 0.05 were considered statistically significant.

## Results

### Specific uptake of ^18^F-FDS by Enterobacteriaceae

**Figure 2:**
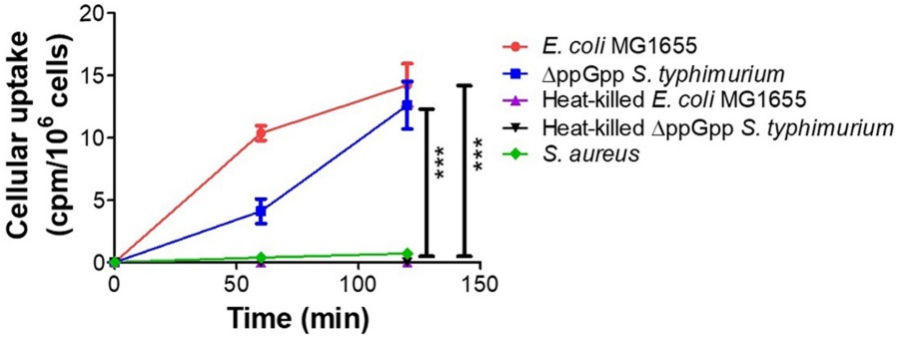
***In vitro* uptake of ^18^F- FDS in bacteria.** *E. coli* MG1655, ΔppGpp *S. typhimurium*, heat-killed *E. coli* MG 1655, heat-killed ΔppGpp *S. typhimurium*, and *S. aureus* cultures were incubated with ^18^F-FDS. *P* values for comparison with the control *S. aureus* were determined by two-tailed Student's *t*-test. *** *p* < 0.001.

We assessed *in vitro* uptake of ^18^F-FDS in cultures of *E. coli* MG1655, ΔppGpp *S. typhimurium*, heat-killed *E. coli* MG 1655, heat-killed ΔppGpp *S. typhimurium*, and *S. aureus*. The two live Enterobacteriaceae species, *E. coli* and ΔppGpp *S. typhimurium*, accumulated ^18^F-FDS, while *S. aureus*, heat-killed *E. coli* MG 1655, and heat-killed ΔppGpp *S. typhimurium* did not (Figure 2).

To test whether the *in vivo* ^18^F-FDS PET imaging could distinguish *E. coli* infection from tumor and sterile inflammation, we inoculated live *E. coli* ATCC 25922 (1 × 10^7^ CFU) into the left thigh, a 10-fold higher burden of heat-killed *E. coli* into the right thigh, and MC38 cells into the right shoulder. Left thigh infections and right thigh sterile inflammations were developed for 10 h before microPET imaging. The PET signals in the infected site were 3.1-fold higher than those in the tumors (*p* < 0.01) and 8.4-fold higher than those in the sterile inflamed site (*p* < 0.001; Figure S1A-B). The correlation between PET SUV_max_ and the number of viable bacteria in the infected left thigh was high (Figure S1C). *E. coli* on the infected thigh showed no translocation to the grafted tumor in the upper part of the body within 10 h. Tumors were harvested for viable bacterial counting immediately after PET imaging, which revealed that the number of bacteria within the tumors was zero in all mice.

### ^18^F-FDS microPET imaging of tumor colonized by *E. coli*

**Figure 3:**
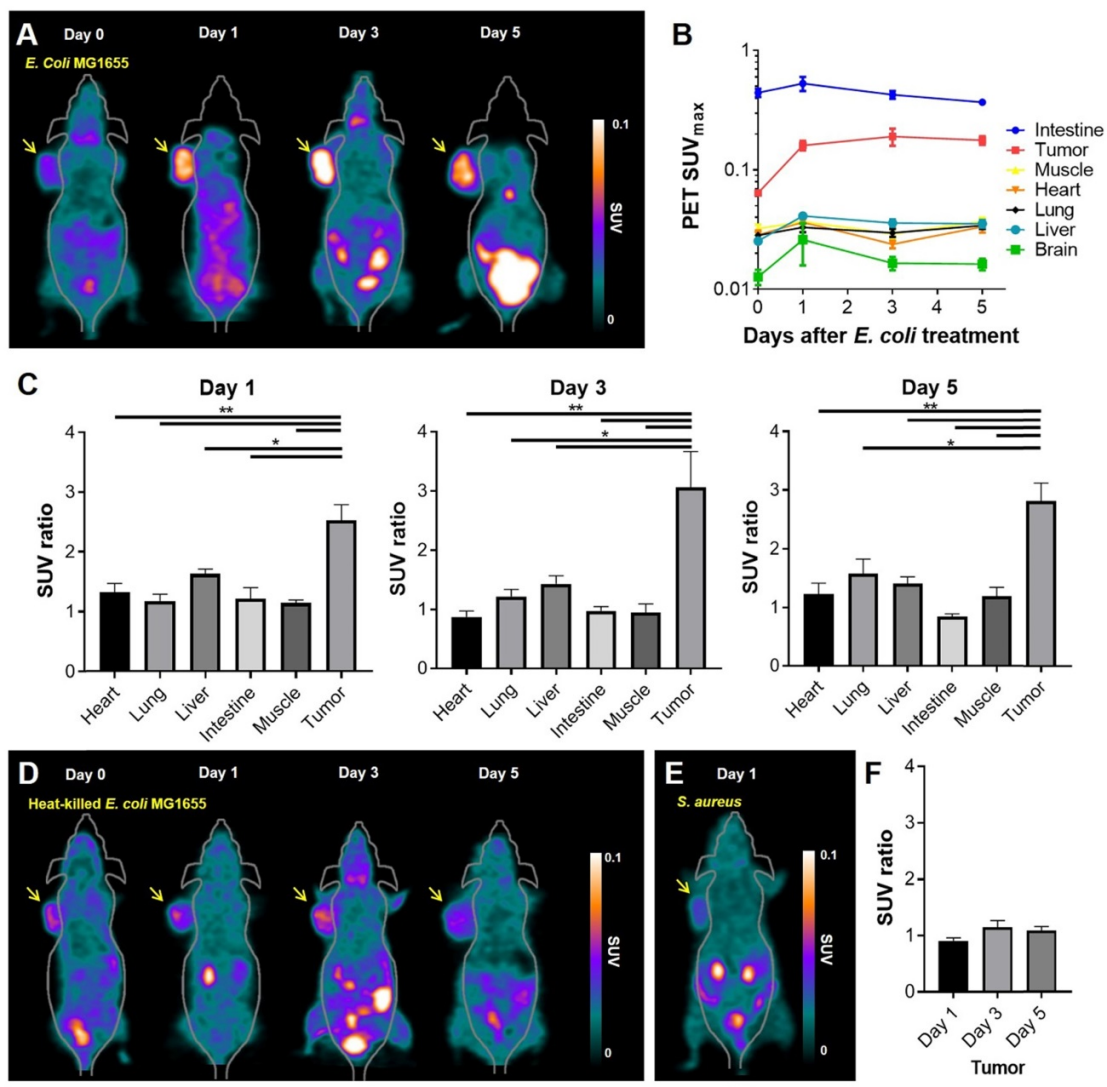
**^18^F-FDS PET imaging of tumor-bearing mice treated with *E. coli* MG1655, heat-killed *E. coli* MG1655, or *S. aureus*. (A)** ^18^F-FDS PET was performed before and 1, 3, and 5 days after intravenous injection of *E. coli* MG1655 (5 × 10^7^ CFU) into subcutaneous CT26 tumor-bearing BALB/c mice. Tumors were harvested for viable bacterial counting immediately after PET imaging. A total of 20 mice were tested, five for each time point. Representative *in vivo* ^18^F-FDS PET images of CT-26-bearing mice. The arrows indicate the locations of engrafted tumors. **(B)** PET signals (SUV_max_) at each time point in the engrafted tumors and normal organs (intestine, muscle, heart, lung, liver, and brain) of data from A. **(C)** SUV ratios obtained as the SUV_max_ of post-bacterial injection divided by the SUV_max_ of pre-bacterial injection in normal organs and engrafted tumors on 1, 3, and 5 dpi (data taken from B). **(D)** Representative ^18^F-FDS PET images performed before and 1, 3, and 5 days after intratumoral injection of heat-killed *E. coli* MG1655 (5 × 10^7^ CFU) into subcutaneous CT26 tumor-bearing BALB/c mice. Engrafted tumors (arrows) were harvested for viable bacterial counting immediately after PET imaging at 5 dpi, which revealed no bacterial colonization of tumors (n = 3). **(E)** A representative ^18^F-FDS PET image taken 1 day after intratumoral injection of *S. aureus* into subcutaneous CT26 tumor-bearing BALB/c mice (n = 3). Engrafted tumors (arrow) were harvested for viable bacterial counting immediately after PET imaging at 1 dpi. **(F)** SUV ratios in engrafted tumors of data from D. *, *P* < 0.05; **, *P* < 0.01. SUV: standardized uptake value.

As the *in vitro* and *in vivo* tests showed^ 18^F-FDS to be specifically concentrated in *E. coli*, we evaluated whether ^18^F-FDS PET could successfully image the colonization of tumor by tumor-targeting *E. coli*. We used ^18^F-FDS PET to measure radioactive signals from tumors and normal organs before and after injection of *E. coli*. ^18^F-FDS uptake in normal organs did not significantly differ between the pre- and post-treatment images (heart, *p* = 0.145; lung, *p* = 0.323; brain, *p* = 0.145; muscle, *p* = 0.118; intestine,* p* = 0.266) except for the liver (*p* = 0.005). Conversely, tumor ^18^F-FDS uptake was significantly higher in 1, 3, and 5 dpi images than in pre-treatment images (*p* < 0.001, each; Figure 3A-B). Liver ^18^F-FDS uptake was also higher in post-treatment images (1.6-fold at 1 dpi, 1.4-fold at 3 dpi, and 1.4-fold at 5 dpi) than in pre-treatment images. These results are consistent with previous reports showing that the number of tumor-targeting bacteria is initially high in the liver and then decreases drastically at 3 to 4 dpi 27, 28.

To further analyze the *E. coli*-specific accumulation of ^18^F-FDS in tumor, we calculated the SUV ratios (SUV_max_ in post-bacterial injection PET)/(SUV_max_ in pre-bacterial injection PET) of tumors and normal organs. As it is known that ^18^F-FDS shows slight accumulation in uninfected tumor due to passive diffusion 22, 24, it was necessary to confirm that ^18^F-FDS accumulated more in colonized tumors than in noncolonized tumors. The SUV ratio was significantly higher in tumors than in the normal organs at 1, 3, and 5 dpi (*p* < 0.05; Figure 3C). While the SUV ratio did not change significantly with time in the normal organs, it tended to increase gradually towards 3 dpi in the colonized tumors (2.5 at 1 dpi, 3.1 at 3 dpi, and 2.8 at 5 dpi). These results are consistent with previous publications, which found the highest numbers of tumor-targeting bacteria in tumor tissue at 3 dpi 27, 28.

Heat-killed *E. coli* MG1655 or *S. aureus* were employed as negative controls of ^18^F-FDS PET imaging. As heat-killed *E. coli* MG1655 or *S. aureus* revealed no tumor tropism, the bacteria were intratumorally injected into subcutaneous CT26 tumors and microPET was performed before and after bacterial injection. MicroPET imaging demonstrated nearly background uptake in tumors injected with heat-killed *E. coli* MG1655 (Figure 3D) or *S. aureus* (Figure 3E). The tumor SUV ratio was significantly lower in tumors colonized by heat-killed *E. coli* MG1655 than in those colonized by live *E. coli* MG1655 (*p* = 0.036) (Figure 3F). The tumor-to-muscle ratio was significantly lower in *S. aureus* treated mice than in live *E. coli* MG1655 treated mice (1.9 ± 0.4 vs. 4.6 ± 1.7, *p* = 0.036). A therapeutic effect was observed only in mice treated with live *E. coli* MG1655 from 3 dpi, but the tumors regrew later (Figure S2).

**Figure 4:**
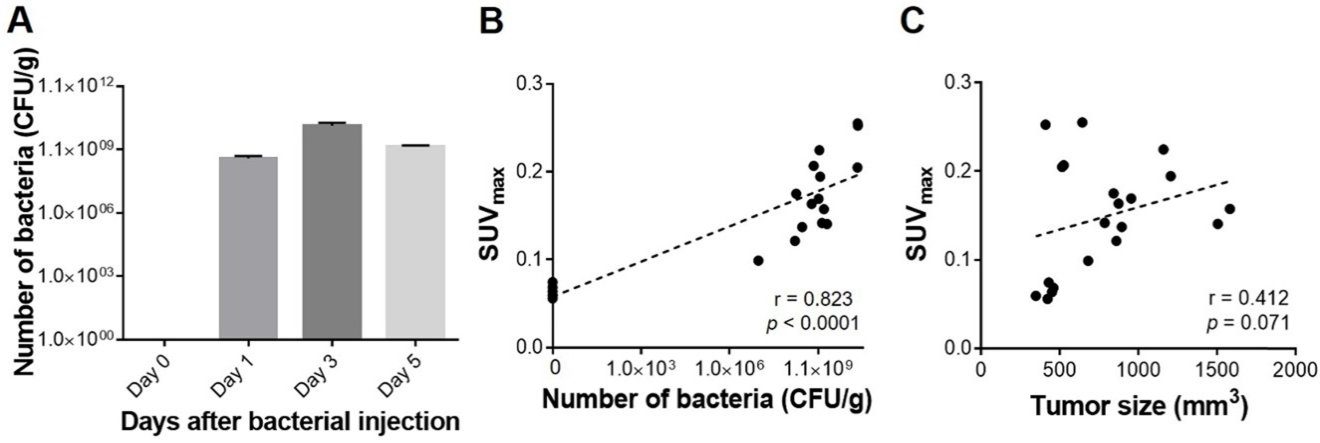
** Quantitative assessment of tumor-colonizing bacteria and PET signals in engrafted tumors. (A)** Viable bacterial counts in harvested tumors.** (B)** Correlation between SUV_max_ and the number of viable bacteria in tumors. **(C)** Correlation between SUV_max_ and tumor size. CFU/g: colony forming unit per gram; SUV_max_: maximum standardized uptake value.

To determine whether the ^18^F-FDS uptake semiquantitatively reflected the number of bacteria in a tumor, we analyzed the correlation between SUV_max_ and the number of viable bacteria in tumors, and found a very strong correlation between them (r = 0.823, *p* < 0.0001). The correlation between SUV_max_ and tumor size (mm^3^) was also fairly high, but it did not reach statistical significance (r = 0.412, *p* = 0.071; Figure 4). SUV_mean_ was as performant as SUV_max_ in predicting the number of bacteria in tumors (r = 0.835, *p* < 0.0001) (Figure S3).

To further assess the feasibility of using ^18^F-FDS PET imaging as a whole body tomographic imaging technique for monitoring BCT, we generated orthotopic colon cancer mice with CT26 mouse colon cancer cells stably expressing firefly luciferase (CT26-Fluc) and treated them with engineered *E. coli* expressing anticancer toxin cytolysin A (*E. coli*-clyA) 25. This process was monitored by ^18^F-FDS PET and bioluminescence optical imaging. ^18^F-FDS specifically accumulated in orthotopic colon tumors in *E. coli*-clyA treated mice but not in the control mice (PBS treatment) (*p* = 0.036). Tumoral bioluminescence activity in the *E. coli*-clyA treated mice tended to increase more slowly than that in controls (Figure S4), although the difference was not statistically significant.

### ^18^F-FDS microPET imaging vs. bioluminescence imaging in mice

**Figure 5:**
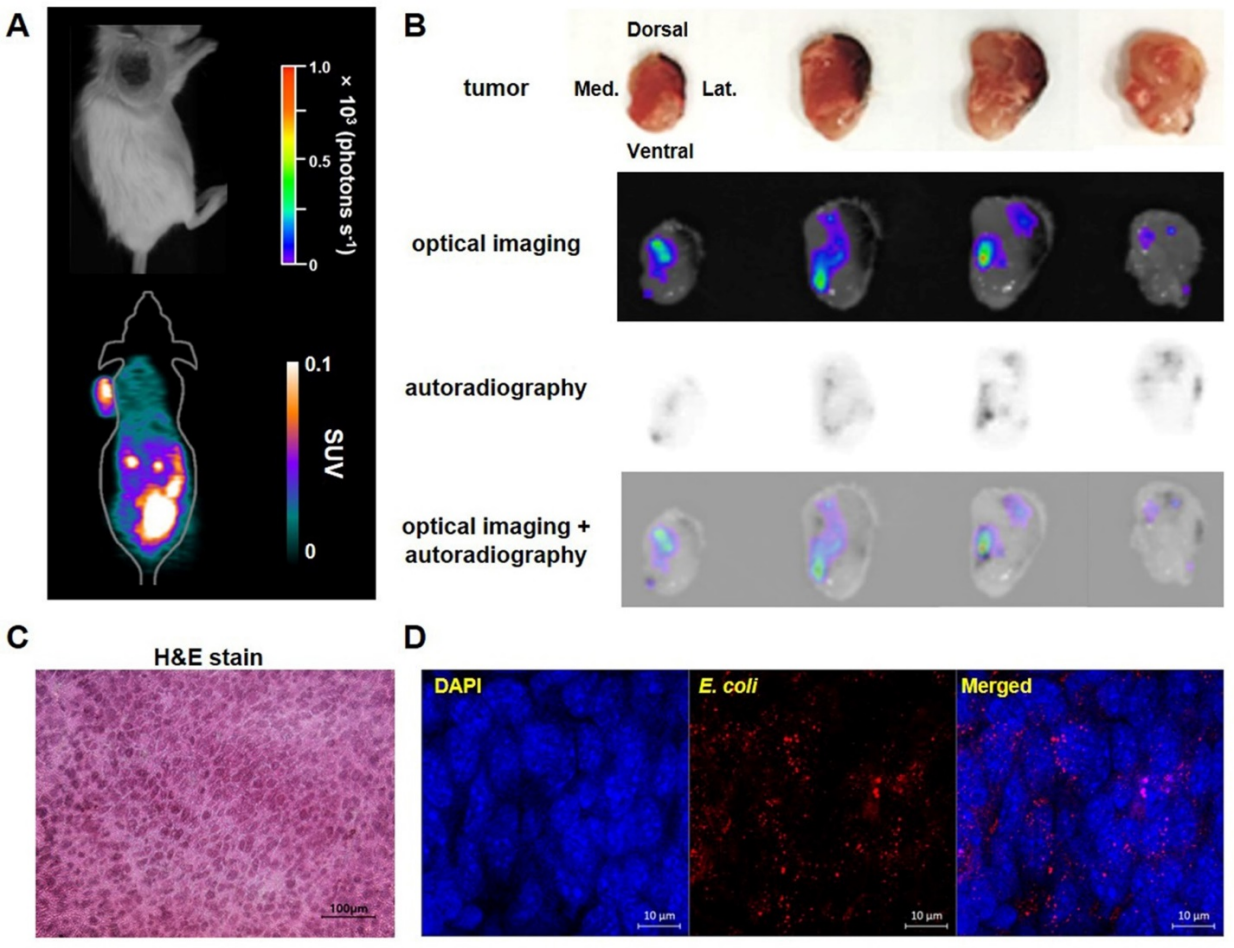
** Bioluminescence imaging and ^18^F-FDS autoradiography of representative tumor-bearing mice injected with *E. coli* expressing lux.** Bioluminescence and autoradiography images were compared using CT26 tumor-bearing mice (n = 4) 3 days after i.v. injection of *E. coli* expressing lux. **(A)** No bioluminescence signal was observed in *in vivo* bioluminescent image, whereas high ^18^F-FDS signal was observed in *in vivo* PET images. **(B)** Ex-vivo photographs, bioluminescence images, ^18^F-FDS autoradiographs, and fused images of bioluminescence images and autoradiographs of 2 mm thick cross sections of the removed tumor. **(C)** Hematoxylin and eosin (H&E) staining of CT26 tumor cells (×400). **(D)** Immunofluorescence staining of tumor tissues. Sections were stained with antibodies against *E. coli* (red). Nuclei were stained with DAPI (blue). A merged image is also shown. Scale bar = 10 µm. H&E: hematoxylin and eosin; SUV: standardized uptake value.

^18^F-FDS PET was more sensitive than bioluminescence imaging in living mice; ^18^F-FDS PET enabled visualization of bacterial colonization from 1 dpi when the number of bacteria was still small, while *in vivo* bioluminescence imaging often failed to visualize bacterial accumulation at this stage (Figure 5A). Moreover, the signal of bioluminescent *E. coli* MG1655 (max radiance) in living mice did not correlate with the number of viable bacteria grown in LB medium (r = -0.115, *p* = 0.751; Figure S5).

To determine whether the locations of ^18^F-FDS uptake in tumors exactly matched with the locations of bacterial colonization, we visually compared ^18^F-FDS autoradiography and bioluminescence images using cross sections of tumors made at 3 dpi. The radioactivity and bioluminescence signals showed similar patterns in most tumor cross sections (Figure 5B). H&E and immunofluorescence staining of tumor cross sections confirmed the presence of tumor tissue and *E. coli*, respectively, in the region corresponding to the foci of high autoradiographic signals (Figure 5C-D).

## Discussion

Despite the rapidly increasing number of published preclinical studies, very few tumor-targeting bacteria treatments have advanced to clinical stages. Although animal models share many genetic elements and biological pathways with humans, fundamental differences still exist. In addition, disease models generally lack the heterogeneity that is always seen in patient populations. The precise *in vivo* localization of bacteria by non-invasive imaging techniques would be of great benefit for preclinical studies, and facilitate the clinical translation of BCT. The ability to image therapeutic bacteria is clinically important because it can provide quantitative data on bacterial density in different body locations in animal models or patients, potentially enabling the prediction of therapeutic response. The bacterial density required to achieve a therapeutic effect does not always correlate with the administered dose because of differences in bacterial proliferation rates in the target tissue. Imaging-based monitoring of the bacterial proliferation could permit non-invasive and repetitive assessment of whether or not an effective bacterial density has been reached in a tumor. As BCT studies predominantly employ Gram-negative Enterobacteriaceae such as *Salmonella* and *E. coli*, ^18^F-FDS, which selectively accumulates in Enterobacteriaceae but not in mammalian or cancer cells, could be widely used as a tracer in BCT studies.

^18^F-FDS PET facilitated successful visualization of tumor-targeting *E. coli* without any need for engineering to create expression of an imaging reporter gene. Two methods that have been used for the PET-detection of bacteria are expression of exogenous viral thymidine kinase 17, 18 and expression of endogenous thymidine kinases 19. When HSV1-TK is expressed in *Salmonella*, it selectively phosphorylates and traps the detectable marker ^124^I-FIAU 18. Alternatively, the endogenous thymidine kinases of *E. coli* Nissle 1917 were shown to phosphorylate and trap ^18^F-FEAU 19. Although both methods have successfully been used to identify bacterial accumulations in mouse tumors, these methods require the synthesis of complicated substrates for the nucleoside-based radiotracers. We here propose using the novel PET tracer ^18^F-FDS to detect bacteria, a tracer that can be easily obtained from the widely available^ 18^F-FDG by simply reducing the aldehyde group to a hydroxyl group 22. Moreover, since ^18^F-FDS has already been used in human clinical trials of Enterobacteriaceae infection 29, ^18^F-FDS PET could facilitate future BCT human trials.

^18^F-FDS PET allowed semiquantitative visualization of bacterial density in tumors. The correlation between ^18^F-FDS PET signals and the numbers of viable bacteria in tumors was strong, while there was no significant correlation between the signal of bioluminescent *E. coli* MG1655 (max radiance) and the number of viable bacteria in tumors. Bioluminescent bacteria can be generated by transforming bacteria with a plasmid encoding bacterial luciferase (pLux); however, bioluminescent bacteria often fail to maintain pLux expression, particularly in infected animals. Therefore, for bacterial transformation, a system that can maintain pLux expression and enable non-invasive quantification of bacterial growth is required, such as the balanced-lethal host-vector system 11, 12.

^18^F-FDS uptake in infected tissues was previously reported to be homogeneous 24. However, in the present study, ^18^F-FDS uptake by cancers colonized by *E. coli* was heterogeneous, which is not surprising because bacterial localization to tumors is known to be heterogeneous 1. Therefore, SUV_mean_ values in colonized tumors might vary depending on the ROI definition. This is why we initially used SUV_max_, which is more reproducible and reliable than SUV_mean_. Nevertheless, in the current study, SUV_mean_ was as effective as SUV_max_ in predicting the number of bacteria in tumors (Figure S3).

^18^F-FDS is known to facilitate bacterial detection when the number of *E. coli* is at least 6.2 ± 0.2 log_10_ CFU 24. In the present study, ^18^F-FDS uptake was well observed in all tumors with bacterial colonization of more than 6.2 ± 0.2 log_10_ CFU. In human clinical trials, the number of bacteria colonizing and proliferating in malignant tissue is expected to be as high as 6.2 ± 0.2 log_10_ CFU 6. Thus, ^18^F-FDS PET imaging should have sufficient sensitivity to visualize the distribution of tumor-targeting bacteria in human studies.

Although ^18^F-FDS revealed basal uptake in tumor and imaging contrast in the subcutaneous U87MG xenografts 22, the ^18^F-FDS accumulation was sufficiently higher in colonized tumors to allow their clear differentiation from uncolonized tumors. Basal tumoral uptake of ^18^F-FDS is unlikely to hamper imaging of bacterial colonization because ^18^F-FDS shows poor intracellular diffusion and retention in tumor cells, but high accumulation in *E. coli* (>1000-fold) 24. ^18^F-FDS, a radiolabeled analogue of sorbitol, is readily taken up by bacteria via a transporter-driven process, phosphorylated, and further metabolized 30. However, in mammalian cells, sorbitol dehydrogenase-mediated retention of ^18^F-FDS is unlikely to be effective because the 2-position of sorbitol is substituted by ^18^F. Without a trapping mechanism for cell retention, cellular uptake of ^18^F-FDS might depend on passive diffusion, driven by the concentration gradient between extra- and intracellular compartments. However, because the hydrophilic character of ^18^F-FDS does not facilitate its penetration of the plasma membrane, it is not retained in the cell 22.

*E. coli* MG1655 was only weakly therapeutic in the current study (Figure S2); therefore, further studies will be required to determine whether PET imaging can be used to visualize the colonization and proliferation of therapeutic bacterial strains and eventually predict therapeutic responses particularly in orthotopic tumor models, such as the one used in the present proof-of-concept study (Figure S4). Previously, we showed that *E. coli* was not as therapeutically effective as attenuated *S. typhimurium* (ΔppGpp strain) 26; therefore, we have begun to study whether ^18^F-FDS PET can visualize ΔppGpp *S. typhimurium* in tumor tissues.

## Conclusions

The results of the present study indicate that ^18^F-FDS PET should have translational power, overcoming the limitations of optical imaging for visualizing and monitoring BCT. ^18^F-FDS PET showed the distribution of tumor-targeting *E. coli*, even in the deep portions of tumors, and provided semiquantitative data on bacterial location without sacrificing the mice. Therefore, ^18^F-FDS PET has potential as a promising *in vivo* imaging technique for visualizing the therapeutic process when BCT is performed with Enterobacteriaceae such as *E. coli*.

## Abbreviations

ΔppGpp: defective in ppGpp synthesis
^18^F: fluorine-18
BCT: bacterial cancer therapy
CFU: colony forming unit
CT26-Fluc: CT26 mouse colon cancer cells expressing firefly luciferase
dpi: days postinoculation
*E. coli*: *Escherichia coli*
*E. coli*-clyA: *E. coli* expressing cytolysin A
FDG: fluorodeoxyglucose
FDS: fluorodeoxysorbitol
FEAU: 1-(2'-Fluoro-2'deoxy-1-β-D-arabinofuranosyl)-5-ethyl-uracil
FIAU: 1-(2'-fluoro-1-β-D-arabino-furanosyl)-5-iodo-uracil
HSV1: herpes simplex virus 1
LB: lysogeny broth
MRI: magnetic resonance imaging
PBS: phosphate-buffered saline
PET: positron emission tomography
pLux: plasmid encoding bacterial luciferase
ROI: region of interest
*S. aureus*: *Staphylococcus aureus*
*S. typhimurium*: *Salmonella typhimurium*
SUV_max_: maximum standardized uptake value
SUV_mean_: mean standardized uptake value
TK: thymidine kinase
