# Supplementary material for: Imaging of tumor colonization by Escherichia coli using 18F-FDS PET
Source: Theranostics. 2020 Apr 1;10(11):4958–66. doi: 10.7150/thno.42121 (PMC7163454; doi:10.7150/thno.42121)
Supplement: Supplementary file 1 — Supplementary figures and tables. [file thnov10p4958s1.pdf]

Supplementary material

Materials and methods

Orthotopic colon cancer model

The orthotopic colon cancer model was established using firefly luciferase-expressing CT26 (CT26-Fluc) murine colon cancer cells. CT26-Fluc cells were injected subcutaneously into the hindlimbs of BALB/c (6 w, female) mice. Two weeks after tumor cell inoculation, a portion (3–4 mm^3) of the harvested subcutaneous tumor was subserosally implanted into the proximal colon of each BALB/c mouse. Subsequently, bioluminescence imaging was performed for about 10 days until the tumor size was approximately 600-800 mm^3. Genetically engineered *E. coli* expressing cytotoxic protein cytolysin A (*E. coli*-clyA) was used for bacterial cancer therapy in this orthotopic tumor model. For tumor-specific therapy with reduced toxicity in normal organs, the L-arabinose-inducible pBAD promoter from the *E. coli* arabinose operon was employed [1]. *In vivo* bioluminescence and ^{18}F-FDS PET imaging were performed before and 3 and 5 days after i.v. injection of *E. coli*-clyA (5×10^7 CFU).

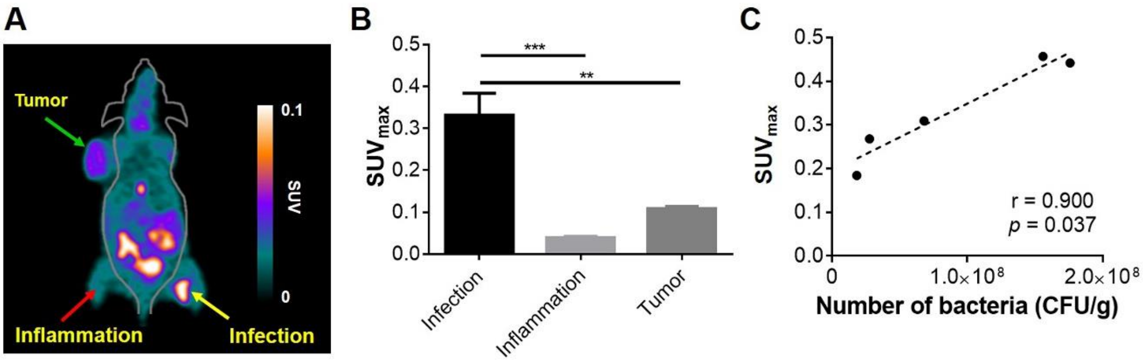

Figure S1. ^{18}F-FDS PET imaging of live *E. coli* infection, sterile inflammation, and tumor. (A–B) A strong ^{18}F-FDS PET signal is visible in the infected thigh (yellow arrows), but the signal is much weaker in the inflamed thigh (red arrow) and the subcutaneous tumor (green arrow). (C) Strong correlation between the SUV_{max} on ^{18}F-FDS PET and the number of viable bacteria in the infected left thigh. **, $P < 0.01$; ***, $P < 0.001$. CFU/g, colony forming unit per gram; SUV_{max}, maximum standard uptake value.

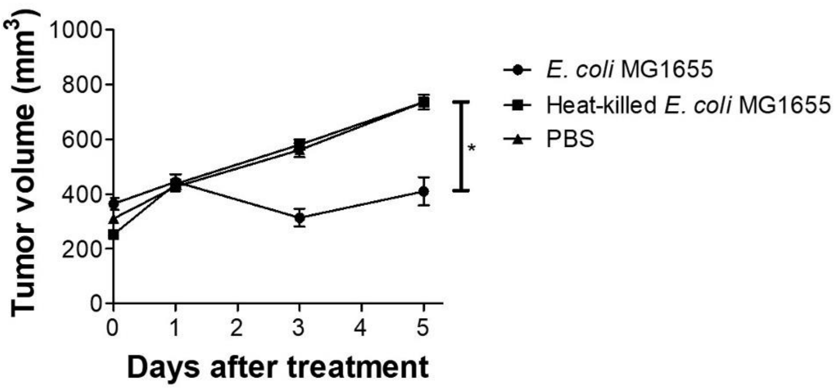

Figure S2. Effects of *E. coli* MG1655, heat-killed *E. coli*, and PBS on tumor volume in a CT26 colon cancer model. In mice treated with *E. coli* MG1655, tumors shrank at 3 dpi but tended to regrow at 5 dpi. * $P < 0.05$. PBS, phosphate-buffered saline.

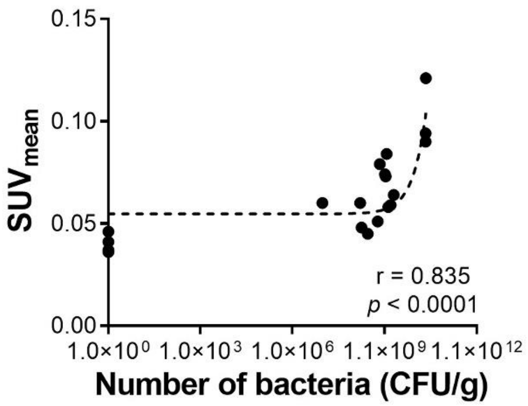

Figure S3. Quantitation of tumor-colonizing bacteria and SUV_{mean} in engrafted tumors. Correlation between SUV_{mean} and the number of viable bacteria in tumors. CFU/g, colony forming unit per gram; SUV_{mean}, mean standardized uptake value.

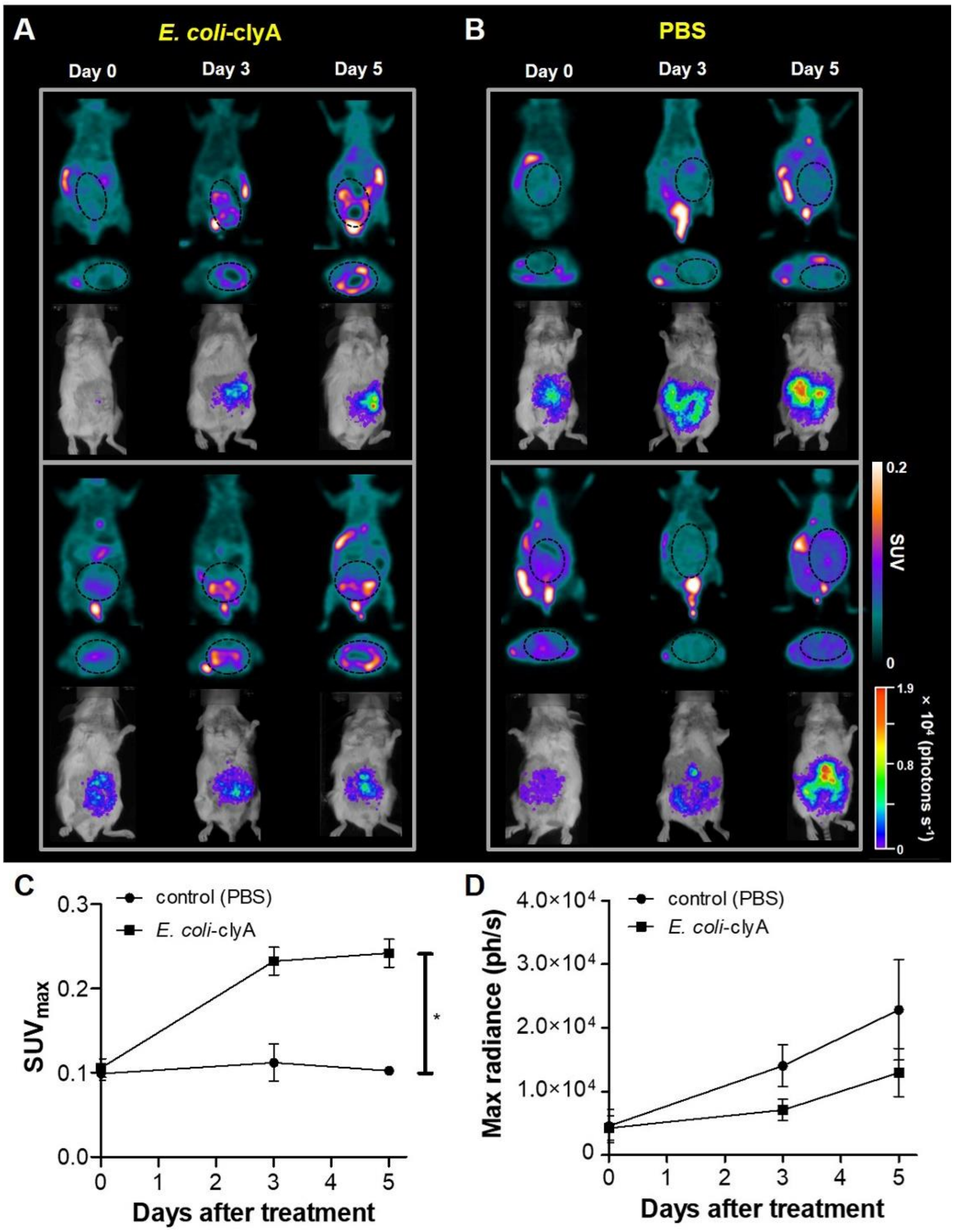

Figure S4. ^{18}F-FDS PET detection of *E. coli*-expressing cytolysin A in the orthotopic colon cancer model. The orthotopic colon cancer model was established using firefly luciferase-expressing CT26 (CT26-Fluc) murine colon cancer cells. CT26-Fluc cells were injected subcutaneously into the hindlimbs of BALB/c (6 w, female) mice. Two weeks after tumor cell inoculation, a portion (3–4 mm^3) of the harvested subcutaneous tumor was subserosally implanted into the proximal colon of each BALB/c mouse. Subsequently, bioluminescence imaging was performed for about 10 days until the tumor size was approximately 600-800 mm^3. Genetically engineered *E. coli* expressing cytotoxic protein cytolysin A (*E. coli*-clyA) was used for bacterial cancer therapy in this orthotopic tumor

model. For tumor-specific therapy with reduced toxicity in normal organs, the L-arabinose-inducible pBAD promoter from the *E. coli* arabinose operon was employed [1]. *In vivo* bioluminescence and ^{18}F-FDS PET imaging were performed before and 3 and 5 days after i.v. injection of *E. coli*-clyA (5×10^7 CFU). ^{18}F-FDS specifically accumulated in orthotopic colon tumors treated with *E. coli*-clyA whereas there was no significant ^{18}F-FDS accumulation in tumors treated with PBS on day 3 ($p = 0.036$) and day 5 ($p = 0.036$) (A-C). Tumoral bioluminescence activity in the *E. coli*-clyA treated mice (D). *, $P < 0.05$. PBS, phosphate-buffered saline; SUV, standardized uptake value.

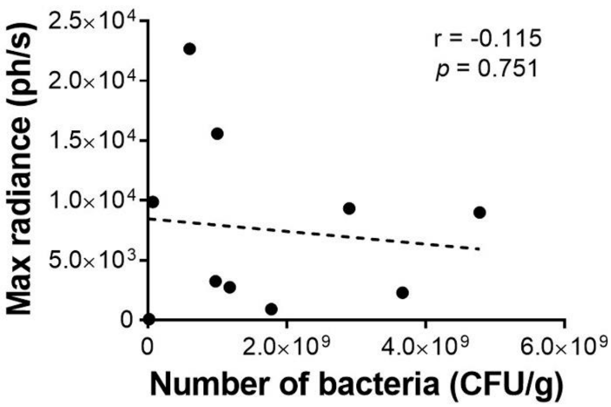

Figure S5. Quantitative assessment of tumor-colonizing *E. coli* MG1655 expressing lux and bioluminescence signals in engrafted tumors. *In vivo* bioluminescence imaging of subcutaneous CT26 tumor-bearing BALB/c mice was performed at 3 dpi of *E. coli* MG1655 expressing lux (5×10^7 CFU). Tumors were harvested for viable bacterial counting immediately after bioluminescence imaging. A total of 10 mice were tested. LB agar plates were inoculated with diluted MG1655 homogenate and incubated overnight at 37 °C. Correlation between max radiance (ph/s) and the number of viable bacteria in tumors was not significant. CFU/g, colony forming unit per gram.

Reference

1. Nguyen VH, Kim HS, Ha JM, Hong Y, Choy HE, Min JJ. Genetically engineered *Salmonella typhimurium* as an imageable therapeutic probe for cancer. Cancer Res. 2010; 70: 18-23.
